# *LIMD1 *is more frequently altered than *RB1 *in head and neck squamous cell carcinoma: clinical and prognostic implications

**DOI:** 10.1186/1476-4598-9-58

**Published:** 2010-03-12

**Authors:** Susmita Ghosh, Amlan Ghosh, Guru P Maiti, Nupur Mukherjee, Sankhadeep Dutta, Anup Roy, Susanta Roychoudhury, Chinmay K Panda

**Affiliations:** 1 Department of Oncogene Regulation, Chittaranjan National Cancer Institute, 37, SP Mukherjee Road, Kolkata 700026, India; 2 Calcutta Medical College and Hospital, 88 College Street, Kolkata-700073, India; 3 Molecular and Human Genetics and Genomic Division, Indian Institute of Chemical Biology, 4 Raja SC Mullick Road, Kolkata 700032, India

## Abstract

**Introduction:**

To understand the role of two interacting proteins LIMD1 and pRB in development of head and neck squamous cell carcinoma (HNSCC), alterations of these genes were analyzed in 25 dysplastic head and neck lesions, 58 primary HNSCC samples and two HNSCC cell lines.

**Methods:**

Deletions of *LIMD1 *and *RB1 *were analyzed along with mutation and promoter methylation analysis of LIMD1. The genotyping of *LIMD1 *linked microsatellite marker, hmlimD1, was done to find out any risk allele. The mRNA expression of *LIMD1 *and *RB1 *were analyzed by Q-PCR. Immunohistochemical analysis of *RB1 *was performed. Alterations of these genes were correlated with different clinicopathological parameters.

**Results:**

High frequency [94% (78/83)] of *LIMD1 *alterations was observed in the samples studied. Compare to frequent deletion and methylation, mutation of *LIMD1 *was increased during tumor progression (*P *= 0.007). Six novel mutations in exon1 and one novel intron4/exon5 splice-junction mutation were detected in *LIMD1 *along with a susceptible hmlimD1 (CA)_20 _allele. Some of these mutations [42% (14/33)] produced non-functional proteins. *RB1 *deletion was infrequent (27%). Highly reduced mRNA expression of *LIMD1 *(25.1 ± 19.04) was seen than *RB1 *(3.8 ± 8.09), concordant to their molecular alterations. The pRB expression supported this data. Tumors with *LIMD1 *alterations in tobacco addicted patients without HPV infection showed poor prognosis. Co-alterations of these genes led the worse patients' outcome.

**Conclusions:**

Our study suggests *LIMD1 *inactivation as primary event than inactivation of *RB1 *in HNSCC development.

## Introduction

Head and neck squamous cell carcinoma (HNSCC) is an aggressive malignancy, accounts for 30-40% of all cancer types in Indian subcontinent [1]. Tobacco, betel nut leaf quid, alcohol, HPV-16/18 infection are well recognized carcinogenic risk factors for development of this cancer [2]. Despite significant progress in understanding molecular genetic events underlying the development of HNSCC, details mechanisms still remain unknown [3,4]. Suppression of tumorigenicity of oral cancer cell lines following introduction of chromosome 3p in microcell hybrid system, suggested the presence of at least one tumor suppressor gene (TSG) in this chromosome associated with HNSCC development [5]. Our previous study in HNSCC of Indian patients showed high frequency of loss of heterozygosity (LOH) in chromosomal (chr.) 3p21.31 region and its association with development of early dysplastic lesions [6]. Among the multiple TSGs localized in chr.3p21.31, our recent study demonstrated one of the candidate TSGs, *LIMD1 *alteration (deletion/methylation) was significantly associated with mild dysplastic lesions of head and neck [7]. Downregulation of this gene observed in HNSCC and lung cancer [7,8]. A recent study emphasized *LIMD1 *as a critical TSG showing frequent downregulation in expression due to genetic and epigenetic modification in human lung cancer [9]. But no coding region mutation of this gene was observed in lung cancer. Also a polymorphic dinucleotide cytosine-adenine [d(CA)] microsatellite repeat, hmlimD1 (Accession number EU125867) was located at 15 bp upstream of *LIMD1 *gene [7]. Susceptibility allele of this gene, if any, for HNSCC development was unknown. *LIMD1 *has 8 exons and encodes a 676 amino acid protein, with a leucine-rich nuclear export signal (NES) in its N-terminal Pre-LIM domain and in C-terminus harboring three LIM domains having nuclear localizing properties (NLS) [8-10]. It is a ZYXIN family protein, having tandem zinc fingers in its LIM domains facilitating protein-protein interactions [11]. LIMD1 was reported to inhibit cell growth and metastases, partly mediated through either an interaction of its N-terminal LEM domain (amino acid 18-68) with barrier-to-autointegration (BAF), a component of SWI/SNF chromatin-remodeling protein, or through interaction of its part of proline-serine rich domain (amino acid 326-608) with C-terminus of retinoblastoma protein, pRB (amino acid 763-928) followed by transcriptional repression of E2F target genes [8]. This might be due to the stabilization of pRB-E2F interaction. The *retinoblastoma *gene, *RB1 *was reported to be infrequently altered in HNSCC [12,13]. Our previous study showed *RB1 *gene deletions were mainly associated with later stages in HNSCC development [14,15]. However, alterations of *LIMD1 *and *RB1 *were not screened in same set of samples to understand their association together in development of the disease.

Thus in this study attempts have been made to analyze the alterations of *LIMD1 *and *RB1 *in 25 dysplastic head and neck lesions, 58 primary HNSCC samples and two HNSCC cell lines. We have screened *LIMD1 *mutation in the entire exon1 (1429 bp) and exon5 along with *RB1 *deletion and its protein expression (by immunohistochemistry, IHC) in the same set of samples. The frequency of LIMD1 mutation was then compiled with our previously reported [7] of its deletion, promoter methylation and mutation (in the SNP rs267236 site in exon1) frequencies in these samples for clinicopathological correlation. The alterations of LIMD1 were also correlated with RB1 alterations. A population-based case-control study was performed to find out any risk allele of hmlimD1. Our data demonstrated that inactivation of *LIMD1 *was primary event than *RB1 *in development of HNSCC.

## Materials and methods

### Patients, controls and cell lines

A total of 37 dysplastic lesions and 110 HNSCC tumors and their matched normal tissues were collected from 147 unrelated individuals after obtaining informed consent from patients according to hospital authorities of Chittaranjan National Cancer Institute and Cancer Center & Welfare Home, Kolkata, India. All tumors were graded and staged according to UICC TNM classification [16]. Freshly operated tissues were taken for isolation of DNA/RNA and immunohistochemical analysis. Among these samples deletion/methylation/mutation analysis of the genes was performed in 25 dysplastic lesions (mild, n = 3; moderate, n = 9; severe, n = 13), 58 HNSCC samples (stageI, n = 8; stageII, n = 11; stageIII, n = 21; stageIV, n = 18) and two HNSCC cell lines Hep2, UPCI: SCC084. However, the total samples pool was used for case-control study. Table 1 presented clinicopathological information of patient (n = 147) and unrelated controls (n = 187) having no previous and present history of HNSCC. Blood samples were collected from controls with informed consent, and Institutional Ethical Board approved the study. All patients and controls were an age and sex frequency-matched ethnically similar caste population from eastern India. Two HNSCC cell lines, Hep2 and UPCI: SCC084 were obtained from National Centre for Cell Sciences, Pune, India and from Prof. Susanne M. Gollin, University of Pittsburgh, USA, respectively.

**Table 1:** Characteristics of patients and controls

Controls(187)		Sex*	
		Male, N(%)	157(83.9%)
		Female,(N%)	30(16.1%)
		
		Mean Age^#^	45.5 ± 5.2
**HNSCC & Dysplastic lesions(147)**		Sex*	
		Male, N(%)	110(74.8%)
		Female,(N%)	37(25.2%)
		
		Mean Age^#^	52 ± 6.4
		
		Lymp Node Positive	26(17.7%)
		
		Lymp Node Negative	121(82.3%)
		
		Tobacco User	103(70%)
		Tobacco free	44(30%)
		
		Alcohol User	13(8.8%)
		Alcohol free	134(91.2%)

**HNSCC (110)**	**Oral Cavity tumors(85)**	Buccal Mucosa, N(%)	32(29.1%)
		Tongue, N(%)	13(11.8%)
		Cheek, N(%)	19(17.3%)
		Lip, N(%)	3(2.7%)
		Tonsil, N(%)	4(3.6%)
		Palate, N(%)	2(1.8%)
		Alveolus, N(%)	11(10%)
		Vocal cord, N(%)	1(0.9%)
	
	**Laryngeal Tumors(12) &**	Larynx, N(%)	12(10.9%)
	**Nasopharyngeal Tumor(3)**	Nassopharynx, N(%)	3(2.7%)
	
	**Orofacial Tumors(10)**	Maxilla, N(%)	5(4.5%)
		Mandible, N(%)	5(4.5%)
	
	**Histopathology(110)**	StageI	15(14%)
		StageII	30(27%)
		StageIII	34(31%)
		StageIV	31(28%)

**Dysplastic lesions (37)**		Buccal Mucosa, N(%)	37(100%)
		
	**Histopathology(37)**	Mild Dysplasia	5(13.5%)
		Moderate Dysplasia	19(51.4%)
		Severe Dysplasia	13(35.1%)

* Difference in sex distribution: Control vs. HNSCC, *P *= 0.04,

^# ^Mean age difference: Control vs. HNSCC, *P *= 0.01.

### Microdissection and DNA Extraction

Cryosections (5 μm) were microdissected under dissecting microscope (Leica MZ16, Germany) using surgical knives to remove contaminant normal cells. Controls' blood and samples containing >60% tumor cells were taken for DNA isolation by phenol/chloroform extraction [17].

### Mutation analysis

*LIMD1 *was screened for mutation in 25 dysplasias, 58 HNSCC samples and the two HNSCC cell lines by single strand conformation polymorphism (SSCP) analysis using [α-P32] dCTP [14]. For mutation analysis six sets of primers were designed to amplify whole exon1 and one set for the exon5 including their respective intron/exon borders (see Additional file 1: Table S1). In these samples deletion and promoter methylation of LIMD1 have already been reported [7], along with mutation status in exon1 at the SNP rs267236 site using *LIMD1 *Exon1.6 primer set (see Additional file 1: Table S1) in the 77/85 samples. Electrophoresis was done in 6% non-denaturing polyacrylamide gel with 10% glycerol at 2W for overnight and autoradiographed on X-ray film (Kodak, USA). Samples showing abnormal band shifts were sequenced using Genetic Analyzer (PE Applied Biosystems Inc, USA).

### Deletion analysis

Deletion mapping of *RB1 *was done in the same set of 25 dysplasias, 58 HNSCC samples and two HNSCC cell lines (as mentioned in **Mutation analysis**) using one intragenic microsatellite marker, D13S153 (intron2 of *RB1*, 47.8 Mb from p-ter) [15].

### Genotyping of hmlimD1 microsatellite marker

Genotyping of hmlimD1 was carried out by amplifying d(CA)_*n *_repeat in a standard polymerase chain reaction (PCR) using [γ-P32] ATP labeled forward primer. PCR products were electrophoresed on 7% denaturing polyacrylamide sequencing gel and autoradiographed [18]. Signal intensities of radio-labeled products were measured by densitometric scanner (Bio-Rad, USA). Amplified fragments ranged in size from 187-216 bp, depending on the number of CA repeats within amplified region. Representative homozygote for (CA)_17_, (CA)_19_, (CA)_20_, (CA)_21_, (CA)_24 _and (CA)_32 _genotypes were sequenced using Genetic Analyzer (PE Applied Biosystems Inc, USA) to validate CA repeats number.

### mRNA expression analysis

The mRNA expression of *LIMD1 *and *RB1 *genes was analyzed by quantitative RT-PCR in paired primary HNSCC samples (n = 24) and two HNSCC cell lines using primers mentioned in Additional file 1: Table S1. The *LIMD1 *mRNA expression was already reported in 11 of the 24 HNSCC samples and the two cell lines [7]. Total RNA was isolated from the samples using TRIzol reagent (Invitrogen, USA) and complementary-DNA (cDNA) was synthesized using Random hexamer (Invitrogen, USA) and M-MuLV Reverse Transcriptase (Sibenzyme, Russia). Real-time quantification of *LIMD1 *and *RB1 *was performed in 40 cycles on an ABI Prism 7500 using Power SYBR Green PCR Master Mix (Applied Biosystems, USA) in a final volume of 25 μl human β2-microglobulin gene (B2M) was used as control. Each sample was loaded in triplicate. Relative level of gene expression was determined by comparative threshold cycle (ddCt) method [19] after normalization against B2M.

### Immunohistochemical analysis

pRB expression was determined by immuno-staining in 5 dysplasias, 15 HNSCC samples and the two cell lines. Paraffin sections of primary tissues and cover slip cultures of the cell lines were reacted with 1:100 dilution of primary antibodies (rabbit polyclonal IgG, sc-7905, for pRB [M-153] Santa Cruz, USA, raised against amino acids 769-921 from C-terminus of pRB). HRP-conjugated (sc-2004 with 1:500 dilutions) and FITC-tagged (sc-2012 with 1:100 dilutions) goat anti-rabbit secondary antibodies (Santa Cruz, USA) were used for the primary tissues and cell lines respectively. For permanent staining of the primary tissues, the slides were developed using 3, 3'diaminobenzidine as the chromogen and counterstained with hematoxylin. The staining intensity (1 = weak, 2 = moderate, 3 = strong) and the percentage of positive cells (<1 = 0, 1-20 = 1, 20-50 = 2, 50-80 = 3 and >80 = 4) were detected by two observers independently and by combining the two scores, final evaluation of expression was done (= 2 = low, 3-4 = intermediate, 5-6 = normal, = 7 = high) [20]. The immunocytochemical stained slides of the cell lines was photographed using fluorescence microscope (Nikon Eclipse E600, Japan).

### Detection of HPV-16 and HPV-18

Presence of HPV in HNSCC was detected by PCR using primers (MY09 and MY11) from consensus L1 region followed by typing of HPV 16/18 in L1 positive samples as described [6].

### Statistical analysis

Fisher's exact test was used to determine different clinico-pathological association with tumors genetic profile, to evaluate case-control difference in distribution of genotypes and to measure the strength of association between *LIMD1 *d(CA)_9-38 _repeat polymorphisms and HNSCC risk. All statistical tests were 2-sided and considered significant at probability value, *P *< 0.05. Survival analysis was performed according to Kaplan-Meier method in 50 HNSCC samples (oral cavity n = 40, orofacial n = 4, larynx n = 4, nasopharynx n = 2). Post-operative overall survival was measured from date of surgery to date of last follow-up or death (upto 5 years). *P*-values were evaluated by log-rank test for censored survival data. Significance and relative risk of various potential prognostic factors was evaluated by Cox proportional hazards model and the hazard ratio (HR) for each parameter with 95% confidence interval (CI) in a multivariate fashion were estimated to predict joint impact of several prognostic factors on overall survival of patients with oral cavity cancer. Analysis of other sites in HNSCC was excluded due to small sample size. All statistical analysis was performed using EpiInfo 6.04b, SPSS 10.0 (SPSS Inc. Chicago, IL, USA).

## Results

### Alterations of *LIMD1*

**Figure 1:**
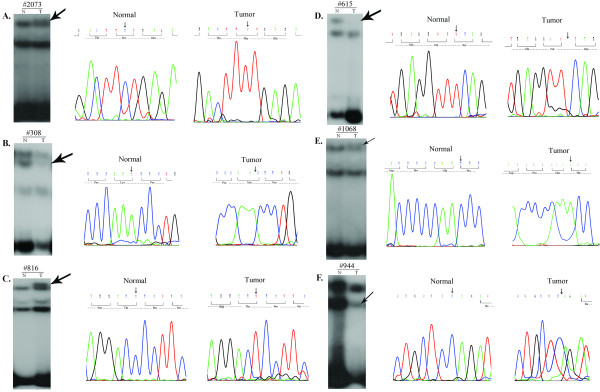
**SSCP autoradiographs and respective chromatographs with resulting aminoacid sequences of representative samples showing mutations in *LIMD1 *exon 1 (A-E), and exon5 (F)**. Arrows indicated shifted bands and nucleotide changes. T, Tumor; N, corresponding normal.

**Figure 2:**
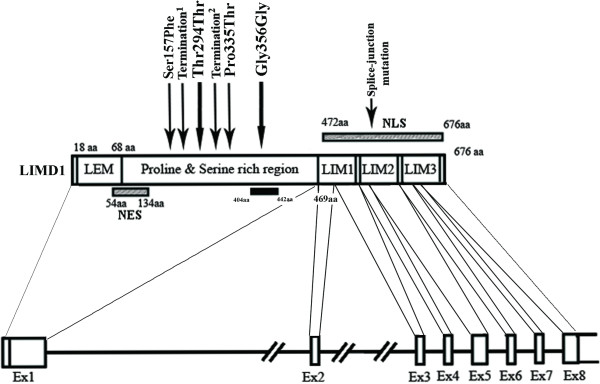
**Schematic diagram of *LIMD1 *gene and protein with mutational hotspot**. Termination^1^,^2^represented premature termination of LIMD1 protein after 7aminoacids and 2 aminoacids respectively. Aminoacids 404-442: Reported pRB binding region [8].

**Figure 3:**
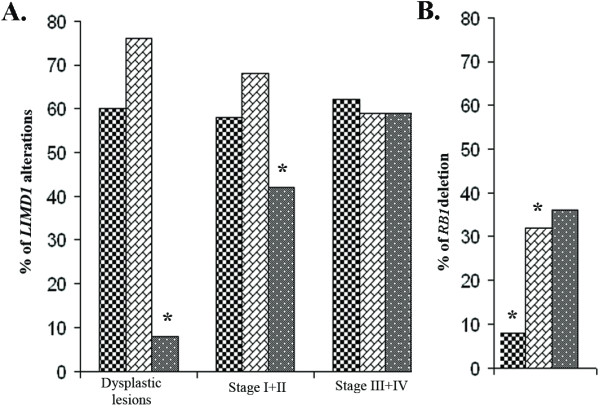
**(A). Patterns of *LIMD1 *molecular alterations during HNSCC progression**. Bars correspond as follows: *: indicated the level of significance. [Checkered line] Deletion; [brick patterned line] Methylation; [grey spotted line] Mutation. **(B)**. *RB1 *deletion pattern in progression of HNSCC. Bars correspond as follows: [Checkered line] Dysplastic lesions; [brick patterned line] Stage I+II; [grey spotted line] StageIII+IV; *: indicated the level of significance.

To prove *LIMD1 *as a candidate TSG, mutation in exon1 and exon5 of this gene was screened in 83 of primary head and neck lesions (25 dysplasias and 58 HNSCC) and two HNSCC cell lines. The deletion and promoter methylation status of *LIMD1 *were screened in these sample previously [7]. Six novel mutations were observed in exon1 (Fig 1A-E) and one was in splice-junction of intron4/exon5 (C deletion 3nt upstream of exon5; Fig 1F, Table 2). Among the mutations in exon1, one transversion with aminoacid changes at 1003C→A (Pro335Thr; Fig 1E), three transition with aminoacid changes at 470C→T (Ser157Phe; Fig 1A) and without aminoacid changes at 882T→C (Thr294Thr; Fig 1C) and 1068T→C (Gly356Gly) [7] and two frameshift mutations due to single nucleotide deletions at 660A (Fig 1B) and 967T (Fig 1D) were observed. Two transition mutations were overlapped with the two SNPs (rs267237 and rs267236) (see additional file 2: Figure S1 and additional file 3: Figure legend for Figure S1.txt). Frameshift mutations led to premature terminations of proteins (Table 2, Fig 2). About 40% (33/83) of the primary head and neck lesions showed mutations in at least one site. Majority of mutations were clustered in proline and serine rich domain of LIMD1 (Fig 2). No mutation was observed in Hep2 and UPCI: SCC084 cells. The status and pattern of LIMD1 alterations (deletion/methylation/mutation) in the 83 of primary head and neck lesions and the two HNSCC cell lines were presented in the additional files (see Additional file 4: Table S2 and Additional file 5: Table S3). Mutation frequency of *LIMD1 *was low (8%, 2/25) in dysplastic lesions, but significantly (*P *= 0.007) increased in stage (I+II) tumors (42%, 8/19) (Fig 3A). Moreover, significant association has been seen between *LIMD1 *mutation and its methylation in HNSCC samples (Table 3).

**Table 2:** Summary of *LIMD1 *mutations

		Mutations						
		
		Exon1						Exon5
		
	Nucleotide change	470C → T	660A del	882C → T	967T del	1003C → A	1068T → C	C del at intron4/exon5
TNM stage	Amino acid change	Ser157 Phe	Frame shift	Thr294Thr	Frame shift	Pro335Thr	Gly356Gly	Splice-juction
								
	Samples showing mutations							
Moderate	L66	-	-	-	-	-	-	-
Moderate	L139	-	-	SNP^a^	-	-	+	-
Severe	L144	-	-	SNP^a^	-	-	-	-
Severe	L126	-	-	SNP^a^	-	-	+	-
Severe	L153	-	-	SNP^a^	-	-	-	-
StageI	#292	-	-	SNP^a^	-	-	-	-
StageI	#2642	-	-	-	-	-	+	-
stageII	#2772	-	-	+	-	-	-	-
stageII	#7216	-	-	-	+	-	-	-
stageII	#2073	+	-	SNP^a^	-	-	+	-
stageII	#5303	-	+	-	-	-	-	-
stageII	#1234	-	-	SNP^a^	-	-	SNP^b^	-
stageII	#2323	-	-	+	-	-	-	-
stageII	#1108	-	-	-	-	-	+	-
stageII	#7077	+	-	-	-	-	-	-
StageIII	#821	-	-	-	-	-	+	-
StageIII	#1367	+	-	-	-	-	-	-
StageIII	#1552	-	-	SNP^a^	-	+	-	-
StageIII	#615	-	-	-	+	-	-	-
StageIII	#5090B	-	-	+	-	-	-	-
StageIII	#816	-	-	+	-	-	-	-
StageIII	#326	-	+	SNP^a^	-	-	-	-
StageIII	#1068	-	-	SNP^a^	-	+	-	-
StageIII	#3893	-	-	-	+	-	-	-
StageIII	#308	-	+	SNP^a^	-	-	-	-
StageIII	#6433	-	+	SNP^a^	-	-	-	-
StageIII	#1332	+	-	SNP^a^	-	-	-	+
StageIII	#2398	-	-	-	+	-	-	-
StageIII	#7059	-	+	-	-	-	-	-
StageIV	#1087	-	-	+	-	-	-	-
StageIV	#4075	-	-	-	-	-	SNP^b^	-
StageIV	#2508	-	+	-	-	-	+	-
StageIV	#5114	-	-	+	-	-	-	-
StageIV	#5219	-	+	-	-	-	-	-
StageIV	#1084	+	-	-	-	-	SNP^b^	-
StageIV	#5184	-	+	-	-	-	-	-
StageIV	#944	-	-	-	-	-	-	+
StageIV	#2785	-	-	SNP^a^	-	-	-	+
StageIV	#1774	-	-	-	-	-	+	-
Cell lines	Hep2	-	-	SNP^a^	-	-	SNP^b^	-
	SCC084	-	-	SNP^a^	-	-	SNP^b^	-

Mutation frequency in HNSCC		6% (5/85)	9% (8/85)	7% (6/85)	4% (4/85)	2% (2/85)	9% (8/85)	4% (3/85)

SNP^a: ^rs267237; SNP^b:^rs267236; del: deletion; L: Dysplastic lesions; #: Invasive samples; '+': Presence; '-': Absence.

**Table 3:** Association study of *LIMD1 *different molecular alterations

	**Dysplastic lesions**			**HNSCC**		
	Del+	Del-	***P***	Del+	Del-	***P***
				
Meth+	**11**	8	0.7022	**21**	15	0.6887
Meth-	4	2		14	8	
			
Mut+	**1**	1	0.7634	**18**	13	0.7036
Mut-	14	9		17	10	
			
	Mut+	Mut-		Mut+	Mut-	
			
Meth+	**1**	18	0.3694	**15**	21	0.02139
Meth-	1	5		16	6	

Del: Deletion; Meth: Methylation; Mut: Mutation; '+': Positive; '-': Negative.

#### Association of hmlimD1 polymorphism with risk of HNSCC

To find out *LIMD1 *susceptible allele, if any, associated with HNSCC risk, we analyzed allele polymorphism of hmlimd1 microsatellite marker in population based case-control study. Age and sex distribution between the cases and controls were found to be statistically significant (Table 1). A total of 12 CA repeat alleles were observed in our study population, ranging from 9 repeats [(CA)_9_] to 38 repeats [(CA)_38_] (Table 4). Among them, alleles (CA)_17 _and (CA)_19 _were relatively common (>10% in frequency) in control population. However, in cases >10% allele frequency was seen in (CA)_19_, (CA)_20 _and (CA)_24 _alleles. Overall, case-control difference in allele distribution was seen to be statistically significant (*P *= 0.0021). Comparing the frequency of each allele with that of all other alleles combined, case-control differences were significant for (CA)_20 _[*P *= 0.000005], (CA)_32 _[*P *= 0.0082] and borderline significance for (CA)_24 _allele [*P *= 0.05427]. The (CA)_20 _allele was over represented in cases, whereas (CA)_17 _and (CA)_19 _alleles were under represented in cases where >10% allele frequency of the alleles were seen. In comparison to homozygous alleles distribution among cases and controls, significant association of [(CA)_20_/(CA)_20_] allele (*P *= 0.00003) was seen with the cases. Alleles (CA)_32 _and (CA)_38 _were rare in this study population.

**Table 4:** Allele frequency of (CA)n polymorphism in the *LIMD1 *gene in head and neck cancer cases and control

Allele	No of (CA)n repeats	Case		Control		***P***^#^
			
		No. of alleles	%	No. of alleles	%	
(CA)_9_	9	7	2.38	11	2.94	0.6571
(CA)_13_	13	4	1.36	35	9.36	0.00012
(CA)_17_	17	23	7.82	46	12.29	0.0592
(CA)_19_	19	97	32.99	169	45.19	0.00139
(CA)_20_	20	66	22.45	36	9.63	**0.000005**
(CA)_21_	21	21	7.14	15	4.01	0.075134
(CA)_24_	24	30	10.2	23	6.15	0.05427
(CA)_26_	26	2	0.68	15	4.01	0.0067
(CA)_27_	27	19	6.46	13	3.48	0.0728
(CA)_30_	30	10	3.4	6	1.6	0.1316
(CA)_32_	32	14	4.76	5	1.34	**0.0082**
(CA)_38_	38	1	0.34	0	0	0.259

Total no. of alleles		294	100	374	100	
		χ^2 ^= 29.175, df = 11, ***P ***= **0.0021**				
	
	Genotype	Case	%	Control	%	***P***^#^
	
	(CA)_17_/(CA)_17_	6	9.23	11	15.94	0.2434
	(CA)_19_/(CA)_19_	33	50.76	56	81.16	0.0002
	(CA)_20_/(CA)_20_	19	29.23	2	2.89	**0.00003**
	(CA)_21_/(CA)_21_	5	7.69	0	0	0.0247
	(CA)_24_/(CA)_24_	1	1.54	0	0	0.4851
	(CA)_32_/(CA)_32_	1	1.54	0	0	0.4851

# From Fisher's exact test.

OR, Odds ratio

CI, Confidence interval

#### Association of *LIMD1 *alterations with *RB1 *deletion in HNSCC

As LIMD1 was shown to interact with pRB [8], we studied the association of alterations of both genes. Compare to high *LIMD1 *alterations, *RB1 *deletion was very low (27%, 22/83) in the primary head and neck lesions (Fig 3B). Deletion frequency of *RB1 *was low in dysplastic lesions (8%, 2/25) and significantly (*P *= 0.045) increased to stage (I+II) tumors (32%, 6/19) consistence with our previous findings [13,14] (Fig 3B). No association between *LIMD1 *alterations and *RB1 *deletion was observed, however co-alteration of both genes was high (33%, 13/39) in the stage III+IV of head and neck lesions (Table 5).

**Table 5:** Association study of *LIMD1 *alterations with *RB1 *deletion

		*LIMD1*					
		
		Dysplasia		Stage I+II		Stage III+IV	
		ALT+	ALT-	ALT+	ALT-	ALT+	ALT-
*RB1*	Del+	2	0	7	0	13	0
	Del-	21	2	11	1	24	2
***P***		0.6637		0.4326		0.3046	

Alt: Overall molecular alterations; Del: Deletion; '+': Positive; '-': Negative.

#### Comparison in the mRNA expression of *LIMD1 *and *RB1*

**Figure 4:**
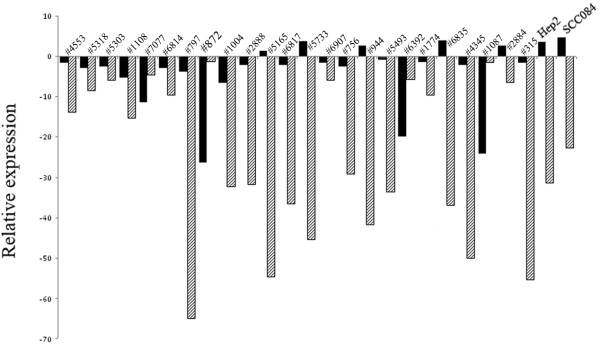
**(A). Quantitative RT-PCR analysis showing *LIMD1 *and *RB1 *expression in HNSCC samples (n = 24), Hep2 and UPCI: SCC084 cell lines**. Bars represented the gene expression normalized to *β2-microglobulin *and relative to normal counterpart. Black bar represented *RB *expression and cross line filled bar represented *LIMD1 *expression.

Quantitative RT-PCR analysis revealed high reduction of *LIMD1 *mRNA expression (25.1 ± 19.04) than *RB1 *(3.8 ± 8.09) (Fig 4). Reduced mRNA expression of LIMD1 showed significant correlation with the gene's methylation (see Additional file 6: Table S4). Half of these tumors (12/24) showed mean fold reduction in *LIMD1 *expression compare to 21% (5/24) in *RB1*. In Hep2 and UPCI: SCC084 cells *RB1 *expression was comparable to the normal tissues than that of reduced *LIMD1 *expression.

#### Immuno-staining of pRB

**Figure 5:**
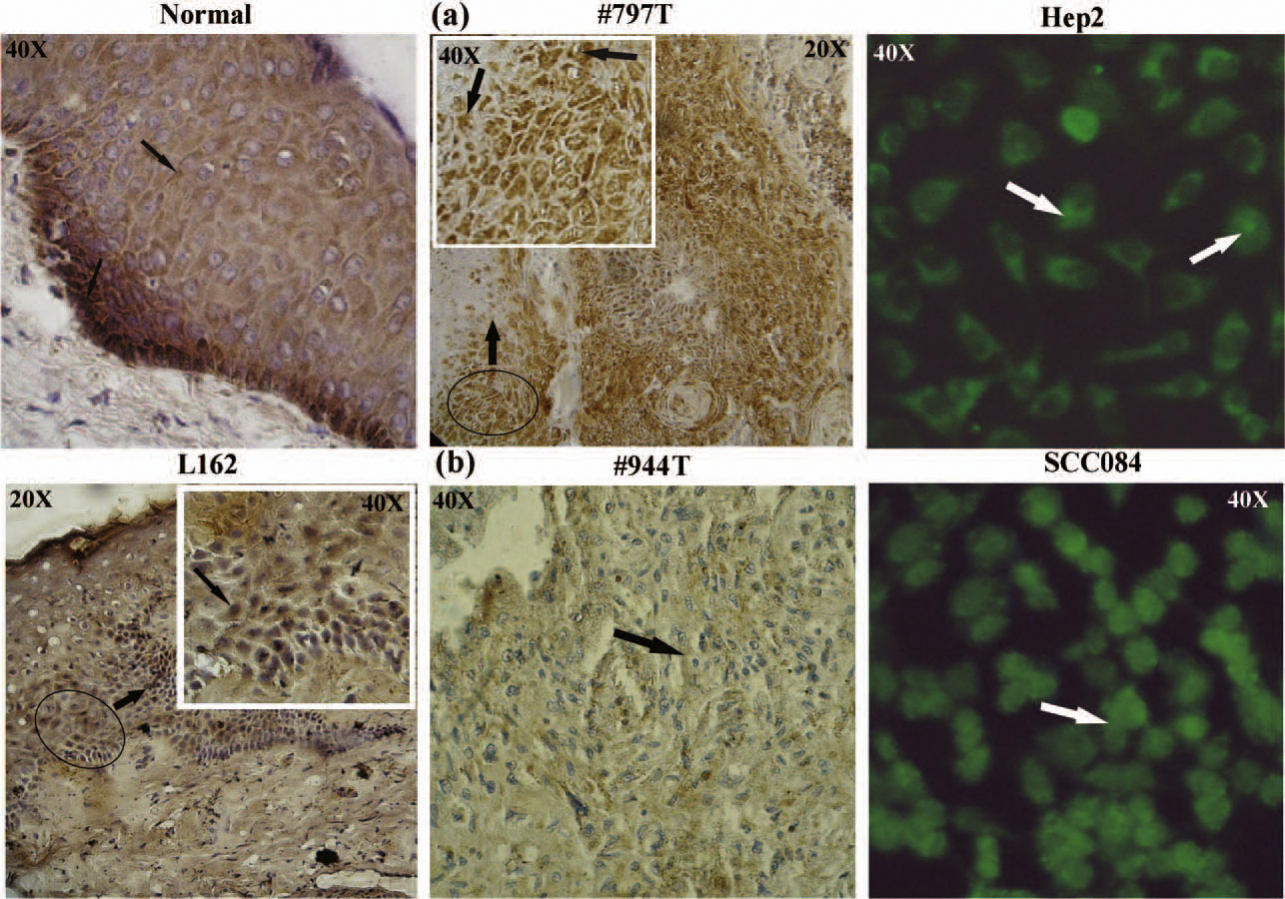
**Immunohistochemical staining patterns of pRB in dysplastic lesions (L), HNSCC samples (T), normal tissues (Normal) and HNSCC cell lines (Hep2, UPCI: SCC084) samples showing high (a), low/negative (b) expression of pRB protein (indicated by arrows)**. In #797T, *RB1 *was not deleted whereas in #944T, this gene was deleted. The regions marked within the circle (20×) were magnified to 40× at inset.

In normal epithelium, immuno-staining revealed intense nuclear and cytoplasmic pRB expression at basal layer, followed by gradual low cytoplasmic expression in differentiated cells (Fig 5). In dysplastic lesions as well as in HNSCC, both nuclear and cytoplasmic expression of pRB was observed. Low/medium level of pRB expression was observed in 30% (6/20) of head and neck lesions contrary to frequent *LIMD1 *alterations (Table 6). In HPV18 positive Hep2 cells, pRB expression was mainly in cytosol, whereas in UPCI: SCC084 (HPV negative), it was expressed both in nucleus and cytoplasm. Concordance was seen between *RB1 *deletion with its RNA and protein expression (Fig 5).

**Table 6:** Correlation between mRNA and protein expression of *RB1 *and their association with *LIMD1 *molecular alterations.

	*RB1*			*LIMD1*	
			
Sample No.; HPV status	Genetic alterations	mRNA status	Protein expression	Genetic/epigenetic alterations	mRNA status
Dysplastic lesions					

L127; HPV-	D-	ND	Normal	D+Me+Mu-	ND

L139; HPV-	D-	ND	Normal	D-Me+Mu-	ND

L154; HPV-	D-	ND	Normal	D+Me+Mu-	ND

L162; HPV-	D-	ND	Normal	D+Me+Mu-	ND

L158; HPV16	D-	ND	Medium	D+Me+Mu-	ND

HNSCC samples					

#1004; HPV-	D-	Normal	Normal	D+Me+Mu-	↓

#5165; HPV-	D-	Normal	Normal	D+Me+Mu-	↓

#6817;HPV16	D+	↓	Medium	D+Me+Mu-	↓

#2884; HPV-	D-	Normal	Normal	D-Me-Mu-	Normal

#1108; HPV -	D+	↓	Medium	D-Me+Mu+	↓

#1774;HPV16	D+	↓	Low	D-Me+Mu+	↓

#872; HPV-	D-	Normal	Normal	D-Me-Mu-	Normal

#5303; HPV-	D-	Normal	Normal	D-Me+Mu+	↓

#6907;HPV16	D-	↓	Low	D-Me+Mu-	↓

#1087; HPV-	D-	Normal	Normal	D-Me-Mu+	Normal

#944; HPV-	D+	↓	Low	D+Me+Mu+	↓

#5733;HPV16	D-	Normal	Normal	D+Me+Mu-	↓

#6392; HPV-	D-	Normal	Normal	D-Me+Mu-	↓

#7077; HPV-	D-	Normal	Normal	D-Me-Mu+	↓

#797; HPV-	D-	Normal	Normal	D+Me+Mu-	↓

Hep2, HPV18	D-	Normal	Normal	D-Me+Mu-	↓

UPCI:SCC084, HPV-	D-	Normal	Normal	D-Me+Mu-	↓

L: Dysplastic lesions; # HNSCC samples; D: Deletion; Me: Methylation; Mu: Mutation; '+': Positive; '-': Negative; '↓': Down expression; ND: Not done.

#### Clinical and prognostic implications of *LIMD1 *and *RB1 *alterations

Using L1 primer, HPV DNA was detected in 56% (82/147) of head and neck lesions of which 84% (69/82) were HPV-16 positive, 11% (9/82) were HPV18 positive and the rest 5% (4/82) were positive for both HPV16/18. Univariate analysis showed significant association of *RB1 *deletion with HPV negativity (*P *= 0.02 for dysplastic lesions; *P *= 0.01 for HNSCC).

**Figure 6:**
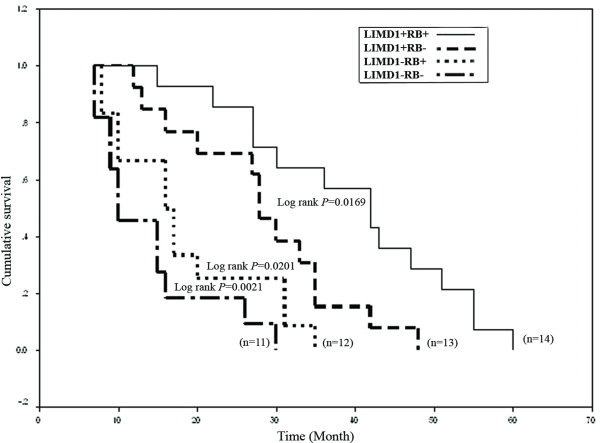
**Kaplan-Meier 5-year survival probability curves with cumulative survival of HNSCC patients by molecular alteration status in *LIMD1 *and *RB1 *loci**. Solid line represented survival probability without molecular alterations of these two genes (wild type, denoted as '+') and dashed line represented the same probability with molecular alterations of either of these genes ('-' indicating altered state). n, total number of HNSCC samples studied in each case.

Log-rank test revealed significant poor survival in patients having alterations in at least one of these genes (Fig 6). Patients with both *LIMD1 *and *RB1 *alterations showed worse prognosis and absence of *RB1 *alterations did not change patients' survival considerably (Fig 6). Multivariate analysis showed that *LIMD1 *molecular alterations (*P*, 0.03; HR, 4.5; CI, 1.1-17.8) along with tobacco addiction (*P*, 0.05; HR, 4.5; CI, 0.96-20.8) in absence of HPV (*P*, 0.05; HR, 0.28; CI, 0.08-0.99) were significant predictor for poor survival of patients with oral cavity cancer (Table 7). Moreover, patients with advanced grade tumor (*P*, 0.50; HR, 1.49; CI, 0.46-4.89) having nodal metastasis (*P*, 0.42; HR, 1.63; CI, 0.49-5.34) along with *RB1 *deletion (*P*, 0.15; HR, 2.27; CI, 0.74-7.02) showed a trend to have poorer survival, albeit being statistically insignificant.

**Table 7:** Multivariate analysis of overall survival of oral cavity cancer patients with different clinicopathological parameters.

Variable	Overall survival		
	
	*P-*value	Hazard ratio(HR)	95% CI for HR
*LIMD1 *alteration	**0.0318**	4.51	1.14-17.82
*RB *deletion	0.1535	2.27	0.74-7.02
Stage	0.5144	0.79	0.39-1.59
Grade	0.5044	1.49	0.46-4.89
Age	0.7786	0.84	0.25-2.85
Node	0.4228	1.63	0.49-5.34
HPV	**0.0498**	0.28	0.08-0.99
Tobacco	**0.0559**	4.49	0.96-20.89
Alcohol	0.2659	0.29	0.03-2.52

CI, Confidence interval.

Bold letters represent significance level.

## Discussion

### Differential alterations of *LIMD1 *and *RB1 *during HNSCC development

To understand the role of *LIMD1 *and *RB1 *in HNSCC development, alterations of these genes were analyzed in 25 dysplastic lesions of head and neck, 58 HNSCC samples and two HNSCC cell lines. In dysplastic lesions *LIMD1 *mutations was less frequent than deletion and methylation (see Additional file 4: Table S2 and Additional file 5: Table S3). However, during progression of the tumor comparable frequencies of these alterations were seen, suggesting the deletion and methylation of LIMD1 as early events in this tumorigenesis. About 80% (67/83) of the samples showed any two of the alterations in *LIMD1 *supporting the modified Knudson two hit hypothesis as a candidate TSG. High frequency (94%, 78/83) of *LIMD1 *alterations (deletion/methylation/mutation) than *RB1 *was seen in the samples. Majority of mutations in *LIMD1 *were present in and around the pRB binding domain of exon1. Unlike us, Huggins *et al. *detected very low frequency (4%, 6/165) of mutation in exon1 of *LIMD1 *at different positions in breast carcinoma [21]. In our samples, about 42% (14/33) of the mutations produced non-functional truncated LIMD1 proteins due to premature termination or splice-junction mutations (Table 2). The significance of synonymous mutations at two SNPs was not clear.

*RB1 *deletion was infrequent with much lower frequency in dysplastic lesions than invasive samples, suggests it less likely to be a candidate TSG in this tumor. Similar to other reports [22], there was a strong association between *RB1 *deletion and reduced pRB expression in our samples (*P *= 0.0006) (Table 6), indicating deletion as the main cause of *RB1 *inactivation. The differential downregulation in mRNA expression of *LIMD1 *and *RB1 *was concordant with their molecular alterations, reflecting genetic imbalances transmitted in transcript level. Intense pRB staining in basal layer of normal epithelial of oral cavity compare to the differentiated cells indicates differential regulation of pRB during differentiation. Similar reports were also found in normal epithelium of esophagus and cervix [23-25]. LIMD1 staining has not been done due to unavailability of commercial antibody. In dysplastic lesions and in HNSCC, pRB expression was seen both in nucleus and cytoplasm where *RB1 *deletion was absent, similar to basal layer of normal epithelium. However the functional status of pRB in tumor cells is not clear. The reduced level of *LIMD1 *in tumor cells might destabilize the pRB-E2F interaction and chromatin remodeling complex, resulting deregulation of cell cycle.

### Identification of susceptible allele of *LIMD1*

As *LIMD1 *has been suggested to be a candidate TSG in HNSCC, our next attempt was to search for a susceptible allele of this gene, if any, associated with HNSCC risk. The case-control study identified (CA)_20 _as the risk allele both in its homozygous and heterozygous state for HNSCC development. Similar repeat length polymorphism in upstream of several genes has been reported [26-28]. The significance of the (CA)_9-38 _repeat variations at upstream of *LIMD1 *was not clear. Regarding the conformation, the (CA)_19 _repeat length might be a critical point. Though the significance of critical (CA)_20 _repeat as risk for this tumor development is not fully understood, it seems that this repeat length might destabilize Z-DNA conformation, causing transcriptional repression of *LIMD1. *Similar effect might also be imposed by (CA)_21_-(CA)_38 _alleles, but due to their low prevalence in our study population, statistical significance has not been observed. In light of the strength of associations and the increased risk for HNSCC, further research is warranted to explore the potential underlying mechanism(s) involved.

### Association of *LIMD1 *and *RB1 *alterations with HNSCC progression and prognosis

Unlike methylation and deletion, significant association of *LIMD1 *mutation with tumor progression indicates that mutation might have some additive effect in inactivation of this gene. In multivariate analysis, association of *LIMD1 *alterations along with tobacco addictions HPV negativity and poor patients' outcome suggests *LIMD1 *as predictive clinical marker in progression of HNSCC. Agreed with similar findings [29-31], significant association was seen in *RB1 *deletion with HPV negative samples and with HNSCC progression. Likewise, a trend towards significance of *RB1 *deletion in advance grade tumors, with nodes of pathology has also been observed [31]. However, worse prognosis of the patients having both *LIMD1 *and *RB1 *alterations suggests *RB1 *inactivation might have some synergistic impact in HNSCC development. Comparatively better survival of patients with *LIMD1*+*RB1*- (*LIMD1 *unaltered &*RB1 *deleted) than *LIMD1*-*RB1*+ (*LIMD1 *altered &*RB1 *unaltered), indicates *LIMD1 *as a key regulator of the disease.

## Conclusion

Thus, it can be concluded that *LIMD1 *is a susceptible gene for HNSCC development and its alterations, alone or with *RB1 *alterations acts as an important prognostic marker in this cancer.

## Abbreviations

HNSCC: Head and neck squamous cell carcinoma.

## Competing interests

The authors declare that they have no competing interests.

## Authors' contributions

SG carried out the molecular genetic studies, participated in the sequence alignment and drafted the manuscript. AG participated in the design of the study and performed the statistical analysis. GM, NM and SD provided the samples needed for this study. AR carried the histological studies and pathological screening. SR participated in its design and coordination and also helped to draft the manuscript. CKP conceived of the study, and participated in its design and coordination and helped to draft the manuscript. All authors have read and approved the final manuscript.

## Supplementary Material

Additional file 1 **Oligonucleotide primers used in the study**. The data report the primers used in the study. Click here for file

Additional file 2 **A representative chromatograph**. The chromatograph represents C/T heterozygous in PBL at SNP rs267237 and C → T mutation in Tumor sample. Click here for file

Additional file 3 **Legend for the Figure S1**. Short explanation for the Figure S1 Click here for file

Additional file 4 **LIMD1 overall molecular alterations including deletion, methylation, mutation and mRNA expression**. The data provided represent the overall molecular alterations ie deletion, promoter methylation, mutation of LIMD1 along with the mRNA expression in each of individual sample studied. Click here for file

Additional file 5 **Pattern of *LIMD1 *alterations in HNSCC during progression of the disease**. The data provided represent the frequencies of deletion, methylation and mutation during the progression of HNSCC. Click here for file

Additional file 6 **Correlation between *LIMD1 *methylation and mRNA expression**. The data provided represent the correlation between LMD1 promoter methylation and its mRNA expression in each individual sample. Click here for file
