# Supplementary material for: LIMD1 is more frequently altered than RB1 in head and neck squamous cell carcinoma: clinical and prognostic implications
Source: Mol Cancer. 2010 Mar 12;9:58. doi: 10.1186/1476-4598-9-58 (PMC2848626; doi:10.1186/1476-4598-9-58)
Supplement: Additional file 1 — Oligonucleotide primers used in the study. The data report the primers used in the study. [file 1476-4598-9-58-S1.DOCX]

Additional file1

**Table S1.** Oligonucleotide primers used in the study

| **Primers** | **Location** | **Analytical purpose** | **Sense** | **Antisense** | **Size (bp)** |
| --- | --- | --- | --- | --- | --- |
| hmlimD1 | -158 to +43 bp ^#^ | Genotyping | 5'-TAGGCAGGTGGAAGTCTTTA-3' | 5’-CCAGGTCGTCATACTTATCC-3’ | 201 |
| *LIMD1* Exon1.1 | Exon 1 | Mutation analysis | 5'-ACACACACACACGGCACCT-3' | 5'-AGGTGGATTTTGGCCATCTT-3' | 217 |
| *LIMD1* Exon1.2 | Exon 1 | Mutation analysis | 5'-AAATCCACCTCCAGCAGCA-3' | 5'-GGTATGGCCTGGATCTCT-3' | 252 |
| *LIMD1* Exon1.3 | Exon 1 | Mutation analysis | 5'-AGCAGAGATCCAGGCCATA-3' | 5'-ACCCACTCCCTACACTCAG-3' | 231 |
| *LIMD1* Exon1.4 | Exon 1 | Mutation analysis | 5'-AGCATCGGCCTGAGTGTAG-3' | 5'-GCTCCGTTCTCCAAGTTT-3' | 257 |
| *LIMD1* Exon1.5 | Exon 1 | Mutation analysis | 5'-ACTTGGAGAACGGAGCACCA-3' | 5'-GCAGAACTGGAAAGGTAAGA-3' | 211 |
| *LIMD1* Exon1.6 | Exon 1 | Mutation analysis | 5'-TCTTACCTTTCCAGTTCTGC-3' | 5'-AGGGGACCCTCTTTACAA-3' | 191 |
| *LIMD1* Exon1.7 | Exon 1 | Mutation analysis | 5'-CCTGCCTGAGTTATCTTGTAA-3' | 5'-AACTCCACCAGCCTCTCACT-3' | 247 |
| *LIMD1* Exon5 | Exon 5 | Mutation analysis | 5'-ACCATCTCATCCTTCCCTAT-3' | 5'-TCCCATCCCTTCTTACTTG-3' | 198 |
| *LIMD1* Exon 1-2 | Coding region of exon | mRNA expression | 5’-GTAAATTCATCGGAGGACCTG-3’ | 5’-CCATCCACAGTCAGCTTG-3’ | 268 |
| *RB* Exon 23-25 | Coding region of exon | mRNA expression | 5’-TTCACCCTTACGGATTCCTG-3’ | 5’-GGTTTAGGAGGGTTGCTTCC-3’ | 230 |
| *B2M* | Coding region of exon | Control for expression | 5’-GTGCTCGCGCTACTCTCTCT-3’ | 5’-TCAATGTCGGATGGATGAAA-3’ | 143 |

# Distance with respect to transcriptional start site.

Microsatellite maker, D13S153 was from Genome Database: Ensembl release 44.

Primer was designed using Primer3Input 0.4.0 software.
