# Supplementary figures and images for: LIMD1 is more frequently altered than RB1 in head and neck squamous cell carcinoma: clinical and prognostic implications
Source: Mol Cancer. 2010 Mar 12;9:58. doi: 10.1186/1476-4598-9-58 (PMC2848626; doi:10.1186/1476-4598-9-58)

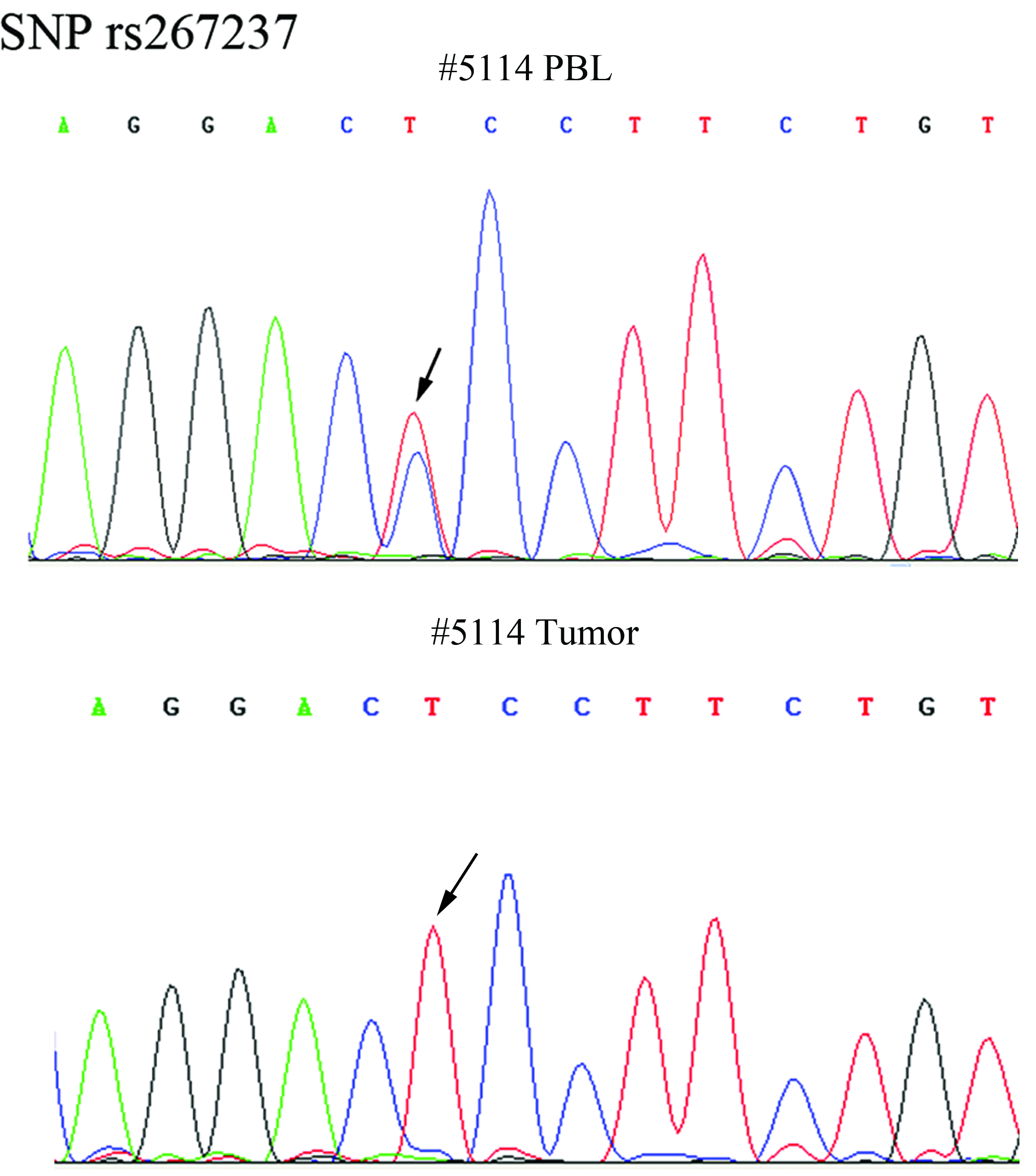

Supplement: Additional file 2 — A representative chromatograph. The chromatograph represents C/T heterozygous in PBL at SNP rs267237 and C → T mutation in Tumor sample. [file 1476-4598-9-58-S2.TIFF]
