# Supplementary material for: LIMD1 is more frequently altered than RB1 in head and neck squamous cell carcinoma: clinical and prognostic implications
Source: Mol Cancer. 2010 Mar 12;9:58. doi: 10.1186/1476-4598-9-58 (PMC2848626; doi:10.1186/1476-4598-9-58)
Supplement: Additional file 3 — Legend for the Figure S1. Short explanation for the Figure S1 [file 1476-4598-9-58-S3.DOCX]

Additional file3

Figure legend for Figure S1: A representative chromatograph showing C/T heterozygous in the SNP rs267237 site in the #5114 PBL (with arrowhead) and C🡪 T mutation in this site in the #5114 tumor.
