# Supplementary material for: LIMD1 is more frequently altered than RB1 in head and neck squamous cell carcinoma: clinical and prognostic implications
Source: Mol Cancer. 2010 Mar 12;9:58. doi: 10.1186/1476-4598-9-58 (PMC2848626; doi:10.1186/1476-4598-9-58)
Supplement: Additional file 5 — Pattern of LIMD1 alterations in HNSCC during progression of the disease. The data provided represent the frequencies of deletion, methylation and mutation during the progression of HNSCC. [file 1476-4598-9-58-S5.DOCX]

Additional file5

**TableS3:** Pattern of *LIMD1* alterations in HNSCC during progression of the disease.

|  | LIMD1 alterations | | |
| --- | --- | --- | --- |
|  | Deletion | Methylation | Mutation |
| Dysplastic lesions | 60% (15/25) | 76% (19/25) | 8% (2/25) |
| StageI+II | 58% (11/19) | 68% (13/19) | 42% (8/19) |
| StageIII+IV | 62% (24/39) | 59% (23/39) | 59% (23/39) |
