# Supplementary material for: LIMD1 is more frequently altered than RB1 in head and neck squamous cell carcinoma: clinical and prognostic implications
Source: Mol Cancer. 2010 Mar 12;9:58. doi: 10.1186/1476-4598-9-58 (PMC2848626; doi:10.1186/1476-4598-9-58)
Supplement: Additional file 6 — Correlation between LIMD1 methylation and mRNA expression. The data provided represent the correlation between LMD1 promoter methylation and its mRNA expression in each individual sample. [file 1476-4598-9-58-S6.DOCX]

Additional file6

**TableS4:** Correlation between *LIMD1* methylation and mRNA expression; ‘↓’ represents the down-regulation of gene expression

| Samples | Methylation | mRNA expression |
| --- | --- | --- |
| #5318 | + | ↓ |
| #2888 | + | ↓ |
| #1004 | + | ↓ |
| #872 | - | Normal |
| #5165 | + | ↓ |
| #6817 | + | ↓ |
| #5303 | + | ↓ |
| #1108 | + | ↓ |
| #6814 | + | ↓ |
| #7077 | - | ↓ |
| #315 | + | ↓ |
| #797 | + | ↓ |
| #6392 | + | ↓ |
| #2884 | - | ↓ |
| #4553 | + | ↓ |
| #6907 | + | ↓ |
| #4345 | + | ↓ |
| #1087 | - | Normal |
| #6835 | + | ↓ |
| #5733 | + | ↓ |
| #5497 | + | ↓ |
| #944 | + | ↓ |
| #1774 | + | ↓ |
| #756 | + | ↓ |
| *P* | 0.00022 | |
